# Methemoglobinemia due to Hemolysis Secondary to Infection in a Child: A Case Report

**DOI:** 10.31729/jnma.7874

**Published:** 2022-10-31

**Authors:** Biraj Parajuli, Swikriti Shrestha, Sandesh Lamichhane, Subash Subedi, Sujata KC

**Affiliations:** 1 Department of Pediatrics, Chitwan Medical College Teaching Hospital, Bharatpur, Chitwan, Nepal; 2 Chitwan Medical College Teaching Hospital, Bharatpur, Chitwan, Nepal

**Keywords:** *case reports*, *hemolysis*, *hypoxia*, *infection*, *methemoglobinemia*

## Abstract

Methemoglobinemia is a rare condition characterised by hypoxic state manifesting as headache, nausea, fatigue, and confusion. We report a 2-year-old boy presenting with fever and cough for 7 days with an episode of hypoxia as the saturation declined and did not improve on face mask oxygenation. On further evaluation, acute intravascular hemolysis was established following decreased haemoglobin level, increased levels of lactate dehydrogenase, and unconjugated bilirubin in the setting of documented infection. Assessment of arterial blood gas showed a significantly raised saturation gap and detection of methemoglobin confirmed the diagnosis. He was managed conservatively with packed red blood cells transfusion following which hypoxia was corrected. Methemoglobinemia as a result of hemolysis can be a non-cardio-respiratory cause of hypoxia and inciting aetiology needs to be addressed.

## INTRODUCTION

Methemoglobin (MetHb) is oxidised haemoglobin in the ferric state that does not bind with oxygen and thus cannot be delivered to the tissues.^1^ A baseline amount of physiologic MetHb formation occurs ubiquitously due to endogenous oxidation in red blood cells. Its formation is regulated via various enzymatic and non-enzymatic processes.^2^ However, this mechanism is overwhelmed in settings of significant oxidative stress resulting in methemoglobinemia.^3^ Only a few studies have reported the co-occurrence of methemoglobinemia and hemolysis after an infection.^3-5^ The clinical presentation of the index child emphasises evaluating non-cardiorespiratory causes of hypoxia in patients presenting with acute hemolysis.

## CASE REPORT

A 2-year boy presented to the Department of Emergency Medicine with fever and cough for 7 days followed by sudden onset pallor and cola-coloured urine for 3 days. On examination, the child was pale, ill-looking, febrile with a respiratory rate of 44 breaths/min, pulse rate of 146 beats/min, blood pressure of 90/50 mmHg, and saturation on pulse oximetry was 80% on room air which did not improve on face mask oxygen at 10 l/min. He had mild tachypnea while the other respiratory findings were unremarkable. He was started on continuous positive airway pressure (CPAP) support at 6 cm of H_2_O but saturation did not improve. On auscultation, lung fields were clear and cardiovascular examination was normal. His arterial blood gas analysis showed pH (7.45), HCO_3_ (18 mmol/l), PO_2_ (144 mmHg), lactate (3.9 mmol/l), SaO_2_ (100%), and the calculated saturation gap of 20% and methemoglobin level was 9.7%. The chest radiograph and 12-lead electrocardiogram were unremarkable thus ruling out cardiac and respiratory causes of hypoxia.

His laboratory findings showed hemoglobin (Hb) (2.8 gm%), white blood cell count (30,300 per mm^3^) (neutrophil 75%), reticulocyte count (11.5%), lactate dehydrogenase (LDH) (5000 U/l), liver function test (LFT), total serum bilirubin (3 mg/dl), direct (0.4 mg/dl), aspartate transaminase (AST) (220 U/l) and alanine transaminase (ALT) (31 U/l) suggesting intravascular hemolysis. Peripheral blood smear showed normochromic normocytic anaemia, glucose 6-phosphate dehydrogenase (G6PD) level of 7.23 U/gHb and a negative Coombs test. Serum procalcitonin (1.8 ng/ml) was raised suggesting an infectious source for which blood culture was sent and was started with empirical intravenous antibiotics. Urine routine examination showed (blood 2+, pus cells 2-4, red blood cells 4-6), no growth in urine culture, and polymerase chain reaction (PCR) for COVID-19 was negative. He was transfused with 2 units of packed red blood cells (PRBC) sequentially and his haemoglobin increased to 9.8 gm%.

As for the cause of hemolysis, a careful review of prescription records showed no offending medications. Methylene blue was not administered as the child clinically improved with PRBC transfusion. His saturation improved to 94% and his urine colour changed to amber on the day of discharge. A repeat hemolytic workup showed improved levels Hb (11.2 g/dl), LFT (total bilirubin= 0.5 mg/dL, direct bilirubin= 0. 1 mg/dl, AST= 45 U/l and ALT= 41 U/l), LDH= 1226 and trace hemoglobinuria. The blood culture showed no growth after 72 hours. Repeat blood gas analysis revealed a methemoglobin level of 1.3% and the patient was discharged on the fourth day of admission.

## DISCUSSION

Methemoglobin is an altered form of haemoglobin in which the ferrous (Fe^2+^) ion is oxidised to its ferric form (Fe^3+^) that does not bind oxygen and as a result, oxygen cannot be delivered to the tissues.^2^ Several mechanisms exist in RBC to reduce MetHb to deoxyhemoglobin to maintain a steady-state level of less than 2% of total Hb. Methemoglobinemia can be either hereditary/genetic (cytochrome b5 reductase deficiency, haemoglobin M, cytochrome b5 deficiency) or acquired after ingestion of drugs like dapsone, antimalarial, topical anaesthetics, and nitrates/nitrites containing food.^2^ Clinical manifestations correlate with the level of methemoglobinemia. Symptoms range from mild cyanosis, dyspnea, or nonspecific symptoms (headache, lightheadedness, fatigue, irritability, lethargy) to shock, severe respiratory depression, or neurologic deterioration (coma, seizures).^6^ The development of cyanosis correlates with the total amount of methemoglobin. Typically, cyanosis occurs if total methemoglobin exceeds 1.5 g/dl. The index case did not have cyanosis at presentation which can be explained due to severe anaemia which masks cyanosis.^3,7^

High levels of methemoglobin can be life-threatening, so early clinical recognition is crucial. A high index of clinical suspicion of methemoglobinemia should be kept in a patient with unexplained hypoxia, not improving with supplemental oxygen, and if the saturation gap is more than 5%. The diagnosis can be confirmed by methemoglobin level in blood gas analysis. Commonly, methemoglobinemia is reported after exposure to oxidant drugs.^8^ In the index child, there was no eliciting medication. Sepsis causing hemolysis and methemoglobinemia has been well reported in the literature.^9,10^

The co-occurrence of methemoglobinemia and hemolysis can occur with infections like hepatitis E, *Campylobacter jejuni* and COVID-19 infection secondary to oxidative stress.^3-5^ We suspect that the index child had suffered from viral prodrome followed by bacterial infection given the history and supporting laboratory findings, but specific focus of infection could not be found. The management of methemoglobinemia is guided by aetiology, severity, and levels of methemoglobin. Discontinuation of the offending drugs or medicines is the most important step for the management along with supportive care for cardiorespiratory and neurological derangements. The symptomatic patients or if methemoglobin level >20% requires methylene blue (1-2 mg/kg intravenously or 100-300 mg orally per day). Ascorbic acid can be used if methylene blue is unavailable or contraindicated (G6PD deficiency as it may precipitate hemolysis).

Blood transfusion and/or exchange transfusion, and hyperbaric oxygen are other treatment modalities that have been reported.^1,11^ In the index case, the possible cause of methemoglobinemia could be acute intravascular hemolysis secondary to infection. In a resource-limited setting like ours, timely identification of methemoglobinemia, evaluating the aetiology and supportive management pose challenges. We administered intravenous antibiotics and transfused packed red blood cells to correct anaemia and the child improved clinically and MetHb levels normalised. Unfortunately, we could not re-evaluate the repeat G6PD level of the index case.

In conclusion, hypoxia not responding to supplemental oxygen therapy with a saturation gap of more than 5% should draw attention to MetHb. Most blood gas analyzer detects MetHb. Symptomatic MetHb due to hemolysis requires supportive management with packed RBC transfusion, identification, and treatment of inciting aetiology.
